# Dietary glycaemic index, glycaemic load and endometrial and ovarian cancer risk: a systematic review and meta-analysis

**DOI:** 10.1038/sj.bjc.6604496

**Published:** 2008-07-29

**Authors:** H G Mulholland, L J Murray, C R Cardwell, M M Cantwell

**Affiliations:** 1 Cancer Epidemiology and Prevention Research Group, Centre for Clinical and Population Sciences, Queens University Belfast, Mulhouse Building, Royal Victoria Hospital Site, Grosvenor Road, Belfast BT12 6BJ, Northern Ireland

**Keywords:** glycaemic index, glycaemic load, endometrial cancer, ovarian cancer, meta-analysis

## Abstract

Long-term consumption of a high glycaemic index (GI) or glycaemic load (GL) diet may lead to chronic hyperinsulinaemia, which is a potential risk factor for cancer. To date, many studies have examined the association between GI, GL and cancer risk, although results have been inconsistent, therefore our objective was to conduct a systematic review of the literature. Medline and Embase were systematically searched using terms for GI, GL and cancer to identify studies published before December 2007. Random effects meta-analyses were performed for endometrial cancer, combining maximally adjusted results that compared risk for those in the highest versus the lowest category of intake. Separate analysis examined risk by body mass index categories. Five studies examining GI and/or GL intake and endometrial cancer risk were identified. Pooled effect estimates for endometrial cancer showed an increased risk for high GL consumers (RR 1.20; 95% CI: 1.06–1.37), further elevated in obese women (RR 1.54; 95% CI: 1.18–2.03). No significant associations were observed for GI. Only two studies examined ovarian cancer and therefore no meta-analysis was performed, but results indicate positive associations for GL also. A high GL, but not a high GI, diet is positively associated with the risk of endometrial cancer, particularly among obese women.

Ovarian and endometrial cancers are currently ranked 7th and 8th of the most common female cancers worldwide, and typically occur in developed countries (WCRF/AICR, 2007). Established risk factors for both cancers include nulliparity and a long lifetime exposure to oestrogen, that is for those who experience early menarche and late menopause, whereas oral contraceptives are known to be protective (Rieck and Fiander, 2006). A high body mass index (BMI) has also been related to ovarian and endometrial cancer risk (Olsen *et al*, 2007; WCRF/AICR, 2007), and risk of the latter cancer can be exacerbated by polycystic ovarian syndrome in overweight or obese women (Furberg and Thune, 2003). Recently, it has been suggested that a moderately high carbohydrate, low glycaemic index (GI) diet may prevent insulin resistance and polycystic ovarian syndrome in women (Marsh and Brand-Miller, 2005).

Dietary GI is a method of classifying carbohydrate quality that ranks foods according to their effect on the 2-h postprandial blood glucose response (Jenkins *et al*, 1981). Food GI calculations are usually based on portions containing 50 g of carbohydrate however an individual's blood glucose, and hence insulin response, varies after consuming differing amounts of carbohydrates. This led to the introduction of the glycaemic load (GL) concept which accounts for the GI and total carbohydrate content of a food and is therefore considered a measure of carbohydrate quality and quantity (Salmeron *et al*, 1997).

Long-term consumption of a high GI or GL diet may lead to chronic hyperinsulinaemia, which has been shown to lower insulin-like growth factor binding protein (IGFBP) concentrations, thereby increasing insulin-like growth factor-1 (IGF-1) levels (Augustin *et al*, 2002). Increased IGF-1 bioactivity inhibits apoptosis, stimulates cell proliferation and sex steroid synthesis and inhibits sex-hormone binding globulin synthesis, all of which could be implicated in the development of endometrial and ovarian cancer (Kaaks *et al*, 2002; Lukanova and Kaaks, 2005).

Consuming a low GI diet may also be beneficial in avoiding weight gain and obesity (Brand-Miller *et al*, 2002), which is a contributing factor in the development of hyperinsulinaemia and excess oestrogen synthesis (Augustin *et al*, 2002). In turn, hyperinsulinaemia, together with boosted plasma IGF-1 levels, are thought to contribute to ovarian hyperandrogenism and a subsequent reduction in endogenous progesterone production, and it has been hypothesised that endometrial cancer risk is increased in women whose oestrogen levels are insufficiently counterbalanced by progesterone (Kaaks and Lukanova, 2001).

The epidemic of obesity, coupled with consumption of high GI or GL diets, are typically associated with developed countries, where the majority of ovarian and endometrial cancers are diagnosed (WCRF/AICR, 2007). In view of the biologically plausible mechanisms suggested above, it could be speculated that dietary GI and GL are associated with endometrial and ovarian cancer development and that BMI is a potential mediator of the effect. To date, studies that have examined the association between GI and GL intake and endometrial or ovarian cancer risk have produced inconsistent results. A systematic review of the literature would therefore be beneficial in order to create evidence-based guidelines for public health.

The aim of the overall systematic review was to establish whether dietary GI and GL are associated with risk of cancer, and in this case, specifically endometrial and ovarian cancer and to examine if risk varies by BMI.

## Materials and methods

### Study selection

Literature searches were conducted using Ovid Medline (US National Library of Medicine, Bethesda, MD, USA), Medline In-Process, Embase (Reed Elsevier PLC, Amsterdam, The Netherlands). The search strategy used medical subject heading (MeSH) terms and keywords: glyc(a)emic index, glyc(a)emic load, blood glucose, blood sugar(s) combined with diet, nutrition, dietary carbohydrate(s), carbohydrate(s), dietary fibre/fibre, fibre/fibre, dietary sugar(s), dietary sucrose and cancer, neoplasm(s), neoplasia, adenoma, adenocarcinoma or carcinoma. Searches were limited to studies conducted on humans published before December 2007. Review publication types were removed but no language restriction was specified. The identified articles were independently screened by two reviewers (HGM and MMC) to determine whether they met the inclusion criteria. To be included, studies with endometrial or ovarian cancer as an outcome had to have measured diet, and subsequently GI and/or GL in participants. The reviewers initially screened abstracts to remove obviously irrelevant articles, and then the full text articles. Discrepancies were resolved by discussion. Finally, the reference lists of all included articles were examined.

### Data extraction

Data extraction was conducted independently by two reviewers (HGM and MMC) using piloted forms to record detailed information on the study design, population characteristics, dietary assessment methods used, confounders measured and, finally, the results. Where essential information was missing, authors were contacted personally. The reviewers applied the Newcastle-Ottawa Quality Assessment Scale (http://www.lri.ca) to all studies to consider factors such as selection of participants, comparability of studies, follow-up and ascertainment of exposure and outcome.

### Statistical analysis

The association between cancer risk and GI or GL intake was summarised by comparing the risk of cancer in the highest reported category of GI (GL) intake to the lowest reported category. Adjusted relative risk estimates (RRs) and their corresponding standard errors were extracted from published reports for each study. The RRs extracted referred to the top quartile or quintile of intake compared with the lowest category of intake. In case–control studies, adjusted odds ratios (ORs) were used, and in cohort studies with variable follow-up: time adjusted hazard rate ratios (HRs) were used, although in some studies these were reported as RRs. Odds ratios and HRs should approximate RRs as endometrial and ovarian cancers are rare (Symons and Moore, 2002). Random effects models were used to calculate pooled RRs, and the *I*^2^ statistic (Higgins *et al*, 2003) was calculated to quantify the degree of heterogeneity between studies. Random effects models were deemed more appropriate than fixed effects models because it was anticipated that there would be study heterogeneity due to their observational nature. Study-specific weights in the random effects model were calculated and scaled to percentages. Publication and selection bias were investigated by checking for asymmetry in the funnel plots of the study RRs against the standard error of the logarithm of the RRs (Sterne and Egger, 2001). The analysis was stratified by BMI, where this information was provided and separate analyses were undertaken for cohort and case–control studies. No meta-analysis was conducted on ovarian cancer because of the small number of studies published. Statistical analysis was conducted using Intercooled STATA (version 9.2, StataCorp 2005, College Station, TX, USA).

## Results

**Figure 1:**
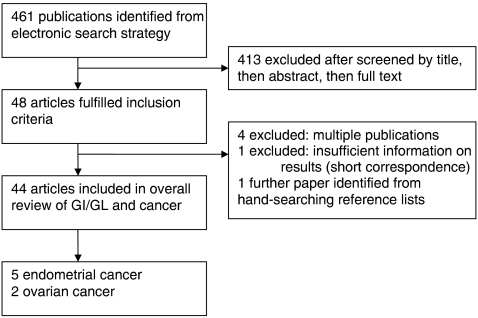
A flow diagram of study selection for GI/GL and endometrial and ovarian cancer risk.

The search strategy results are shown in Figure 1. Five articles that were identified investigated endometrial cancer (Folsom *et al*, 2003; Augustin *et al*, 2003a; Silvera *et al*, 2005; Larsson *et al*, 2007; Cust *et al*, 2007a) and two that investigated ovarian cancer (Augustin *et al*, 2003b; Silvera *et al*, 2007); the characteristics of these studies are described in Table 1.

All studies principally employed either self-reported or interviewer-administrated validated Food Frequency Questionnaires (FFQs). Glycaemic index/GL values were primarily sourced from International Tables (Foster-Powell and Miller, 1995; Foster-Powell *et al*, 2002; Henry *et al*, 2005; University of Sydney website), with some studies supplementing these with local sources (Augustin *et al*, 2003b). All studies were conducted in Europe or North America. Many potential confounders were adjusted for in individual publications but these were inconsistent between studies (Table 1). All adjusted for age and energy intake, whereas adjustments for hormonal use, reproductive factors, menstrual history, physical activity and other dietary variables including alcohol intake varied between studies.

### Endometrial cancer

Prospective cohorts accounted for four of the five studies identified that examined endometrial cancer risk, all of which incorporated adequate follow-up lengths, whereas the remaining study was a hospital-based case–control study. Cohort studies scored more highly on the quality scale compared with the case–control study (Table 1). In all studies, cases were identified by microscopic verification, linkage to cancer registries or self-report and women who had undergone a hysterectomy were excluded.

**Figure 2:**
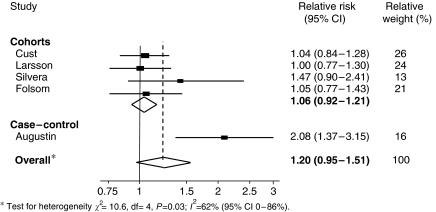
Meta-analysis of GI and endometrial cancer risk.

The association between endometrial cancer and GI intake for all five studies is shown in Figure 2. The combined adjusted RR of endometrial cancer in the highest reported category of GI intake compared with the lowest reported GI category was 1.20 (95% CI: 0.95–1.51); however, there was evidence of statistical heterogeneity (*I*^2^=62%, *P*=0.03). The statistical heterogeneity was markedly reduced (*I*^2^=0%, *P*=0.69) when only the four cohort studies were included in the analysis. There was little evidence of an association between GI and endometrial cancer in these studies (RR 1.06; 95% CI: 0.92–1.21).

**Figure 3:**
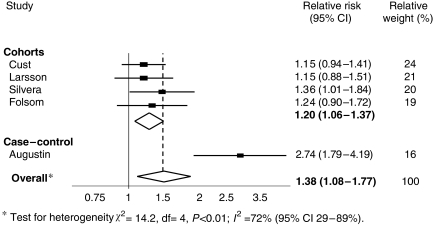
Meta-analysis of GL and endometrial cancer risk.

The association between endometrial cancer risk and GL intake for the five studies is shown in Figure 3. The combined adjusted RR of endometrial cancer in the highest reported category of GL intake compared with the lowest reported category of GL intake was 1.38 (95% CI: 1.08–1.77), but there was also evidence of marked heterogeneity (*I*^2^=72%, *P*<0.01). Again, after the removal of the case–control study, this heterogeneity was markedly reduced (*I*^2^=0%, *P*=0.80). The combined adjusted RR for the four remaining cohort studies was still statistically significant showing a 20% increased risk for endometrial cancer in women consuming a high GL diet (RR 1.20; 95% CI: 1.06–1.37). The European Prospective Investigation into Cancer and Nutrition (EPIC) study also reported results of GI and GL intake as continuous variables and showed an elevated risk of endometrial cancer of 1.40 (95% CI: 0.99–1.99) per 50 U day^−1^ increments in GL. There was little evidence of the presence of publication bias from examining funnel plots for GI or GL and for endometrial cancer risk.

**Figure 4:**
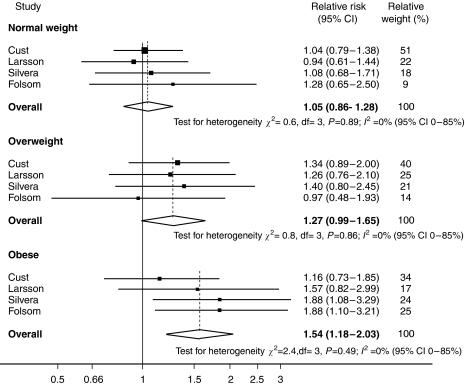
Meta-analysis of GL and endometrial cancer risk by BMI categories: normal weight (BMI < 25 kg/m^2^), overweight (BMI ⩾25 and <30 kg/m^2^) and obese (BMI ⩾30 kg/m^2^).

The association between GL and endometrial cancer after stratification by BMI is shown in Figure 4. Additional information by BMI stratification was sought from Cust *et al* (2007a), as this study had indicated that the analysis had been conducted but not published. Stratified analysis in the case–control study was only shown for GI intake and additional information for GL was not obtained due to variation in BMI cutoffs. There was little evidence of an association between GL and endometrial cancer risk in the normal weight women (RR 1.05; 95% CI: 0.86–1.28), some evidence of increased risk in the overweight group (RR 1.27; 95% CI: 0.99–1.65) and strong evidence of a positive association in obese women (RR 1.54; 95% CI: 1.18–2.03). There was little evidence of heterogeneity between studies in the analysis stratified by BMI. No significant associations were observed when GI and endometrial cancer risk were investigated by BMI category, with RRs of 1.03 (95% CI: 0.81–1.31), 1.42 (95% CI: 0.95–2.11) and 1.01 (95% CI: 0.71–1.43) detected for normal weight, overweight and obese women, respectively.

Stratified analyses were performed according to other variables; however, too few studies did so to enable robust investigation of these by meta-analysis. Conflicting results indicated that the association between GL and endometrial cancer risk may be influenced by menopausal status (Silvera *et al*, 2005; Cust *et al*, 2007a), hormone replacement therapy (HRT) use (Augustin *et al*, 2003a; Folsom *et al*, 2003; Silvera *et al*, 2005), physical activity levels (Silvera *et al*, 2005; Larsson *et al*, 2007) or diabetes history (Folsom *et al*, 2003).

### Ovarian cancer

One prospective cohort and one large case–control study of GI and GL intake and ovarian cancer risk have been conducted to date, with the latter study scoring slightly lower on the quality scale (Table 1). The cohort recruited women as part of the Canadian National Breast Screening programme, identifying cases by record linkage to national cancer and mortality databases. Although the Italian case–control study recruited hospital-based controls who may not reflect the general population, cases were histologically confirmed and any controls with modified dietary habits were excluded. Notably, although the latter study failed to adjust for BMI in their main analyses (Table 1), subgroup analyses results were stratified by BMI in the original publication.

Both studies detected an increase in the risk of ovarian cancer in the highest reported GI category compared with the lowest, although only the case–control study detected this association as significant (OR 1.65; 95% CI: 1.30–2.09). Stratified analyses within the latter study demonstrated stronger associations between GI and ovarian cancer risk in postmenopausal women (which was not observed in the cohort study), overweight women, non-diabetics, non-oral contraceptive users, parous women, alcohol consumers and women without a family history of breast and/or ovarian cancer. Highly significant increased risks were detected in both studies of ovarian cancer and a high GL diet in all women (HR 2.15; 95% CI: 1.29–2.09 and OR 1.65; 95% CI: 1.30–2.09, respectively). Both studies acknowledged that the majority of cases identified were postmenopausal and associations tended to be stronger for postmenopausal compared with that for premenopausal women.

## Discussion

This is the first systematic review of the evidence to date on GI and GL intake and endometrial cancer risk in relation to BMI and provides evidence of a positive association between high GL diets and endometrial cancer risk but little evidence of an association for dietary GI intake. The finding that a high GL diet increases the risk of endometrial cancer compared with a low GL diet demonstrates the advantage of a meta-analysis because only one of the individual studies reported statistically significant results, although all studies showed associations in a positive direction. It should be noted that some individual studies had a relatively narrow range of GI and GL intakes, which could explain the lack of statistically significant results observed in these studies. In the cohort studies, comparing the highest versus the lowest category of GL intake corresponded to an approximate difference of 50 GL units (Folsom *et al*, 2003; Silvera *et al*, 2005, 2007; Cust *et al*, 2007a; Larsson *et al*, 2007), whereas in the case–control studies this observed difference was approximately 100 units (Augustin *et al*, 2003a, 2003b). This could partly explain the stronger association observed in the meta-analysis of endometrial cancer risk and GL when the case–control study was included.

We did not observe an association between dietary GI and endometrial cancer risk. This would suggest that endometrial cancer risk is related to the actual blood glucose, and hence insulin, demand induced by the consumption of normal portion sizes of carbohydrates rather than the standard 50 g used to calculate GI values. Despite this, GI is still an important contributor to the GL value of a food, and it would be preferable to advise individuals to consume a diet composed of low GI/moderate total carbohydrate content as opposed to high GI/low total carbohydrate content to achieve a low dietary GL intake. There is a paucity of research examining the association between dietary factors and endometrial or ovarian cancer risk. Investigations of total carbohydrate or cereals have provided weak or null associations (Jain *et al*, 2000; Trichopoulou *et al*, 2000). Identifying a strong association for any modifiable dietary factor and endometrial or ovarian cancer is therefore extremely valuable. In addition, there is little evidence to suggest that a low GI diet may have any accompanying adverse effects, and thus advising women to consume a low GL diet would seem a reasonable approach (Colombani, 2004).

Previous reports have shown a linear dose–response relationship between BMI and endometrial cancer risk (Jain *et al*, 2000; Furberg and Thune, 2003; Schouten *et al*, 2004; Friedenreich *et al*, 2007). Our findings that a high GL diet increases the risk of endometrial cancer as BMI increases, suggests that BMI may be an effect modifier of the association between GL and endometrial cancer, and that high GL diets may exaggerate endometrial cancer risk in women who are more likely to be insulin resistant. Additionally, a recent meta-analysis of 16 studies demonstrated that diabetics have over twice the risk of developing endometrial cancer compared with that of non-diabetics (Friberg *et al*, 2007). Similar elevated risks of ovarian cancer incidence and mortality were observed in a large UK cohort of diabetics compared with the general population (Swerdlow *et al*, 2005). Consuming low GI carbohydrates has been associated with improved glycaemic control in diabetic patients compared with high GI diets in randomised controlled trials (Brand-Miller *et al*, 2003). Other subgroup analyses in the papers included in our review have suggested that the association between GL and endometrial cancer may be modified by diabetes, menopausal status, HRT use or physical activity (Augustin *et al*, 2003a; Folsom *et al*, 2003; Silvera *et al*, 2005; Cust *et al*, 2007a; Larsson *et al*, 2007). Unfortunately, we were unable to perform meta-analyses on the basis of HRT use, diabetes or physical activity as too few studies reported results by these stratifications, and therefore no conclusions can be drawn with respect to these variables.

Although the mechanisms are recognised to differ by cancer site, the IGF system is often reported as the proposed mediator between GI, and therefore potentially GL and cancer risk (Du *et al*, 2006). A validation study of the effect of GI on the insulin response did report an overall 70% reduced insulin response after consumption of a low GI food compared with a high GI food (Brand-Miller *et al*, 2005). However, alterations to IGF-1 and IGFBP-3 levels were minimal following the low GI food compared with the high GI food. Notably, this study was conducted in lean young subjects, so the application of these findings to obese people is currently unknown. Obese subjects are known to have elevated circulating IGF-1 levels as a result of overnutrition (Augustin *et al*, 2002), and it is therefore plausible that a high GL diet in people with a higher BMI has a more profound effect on IGF-1 levels. Unfortunately, studies that have investigated the association between the IGF system and endometrial and ovarian cancer risk have produced inconsistent findings (Lukanova *et al*, 2004; Peeters *et al*, 2007; Tworoger *et al*, 2007). Despite this, C-peptide, a marker of pancreatic insulin production, has repeatedly been shown to be directly related to endometrial cancer risk in well-designed studies (Lukanova *et al*, 2004; Cust *et al*, 2007b), suggesting a key role for hyperinsulinaemia. The majority of endometrial cancers are oestrogen-related (WCRF/AICR, 2007), therefore hyperinsulinaemia induced by a habitually high GL diet may explain the increased endometrial, and possibly ovarian, cancer risk by the ‘unopposed oestrogen’ hypothesis.

Oestrogen is a known mitogen, and overweight and obesity could increase endometrial and ovarian cancer risk due to ovarian hyperandrogenism (surplus ovarian androgen production), which is promoted by hyperinsulinaemia, resulting in a subsequent oestrogen excess derived from the aromatisation of androgens in adipose tissue (Kaaks *et al*, 2002). In postmenopausal women, the cessation of progesterone synthesis results in oestrogen concentrations being insufficiently counterbalanced, while in hyperinsulinaemic premenopausal women, absent ovulation and ensuing progesterone deficiency may also enhance the mitogenic potential of oestrogen (Kaaks and Lukanova, 2001; Kaaks *et al*, 2002). Others have recognised that insulin levels *per se* cannot explain the disparity in the observed risk between obesity and premenopausal and postmenopausal breast cancer risk, whereas oestrogen concentrations are well correlated (Key, 2001).

The precise mechanisms through which a high GL diet increases endometrial, and potentially ovarian, cancer risk needs to be clarified in further research that utilises independent biomarkers such as C-peptide or components of the IGF system, in addition to dietary exposure. The inclusion of biomarkers could help to overcome the limitations associated with dietary GI and GL evaluation. For example, GI and GL values apply only to single foods, so when composite meals are consumed the ability to predict insulinaemic responses from GI values has been questioned (Flint *et al*, 2004). Blood glucose and insulin concentrations can also be influenced by other dietary components such as protein and fats (Jenkins *et al*, 1984). Furthermore, the studies in our systematic review used a mixture of glucose and white bread reference values for GI and GL. The reproducibility of GI and GL values and their application to different population groups are often highlighted as important methodological issues (Feskens and Du, 2006), and to our knowledge, the validity of combining GI and GL results from different populations is currently unknown.

Other limitations to individual study designs reported in this review include the possibility of recall bias in case–control studies, whereby cases recall their diet differently compared with healthy controls (Augustin *et al*, 2003a, 2003b). Self-administered FFQs were used in all of the prospective cohorts, none of which were specifically designed for assessing GI or GL intake. Food Frequency Questionnaires are known to incorporate some dietary measurement error, especially among overweight or obese individuals but are the most convenient assessment tool available for large-scale studies (Black *et al*, 1993). The FFQ employed in one of the case–control studies was also relatively short, including only 37 items (Augustin *et al*, 2003a). Only two cohorts incorporated repeat dietary measures (Cust *et al*, 2007a; Larsson *et al*, 2007), one of which used a single 24-h recall in a stratified random sample of participants in addition to an FFQ (Cust *et al*, 2007a), whereas the other obtained dietary information using an FFQ at two different time points (Larsson *et al*, 2007). Despite potential dietary measurement error, it is unlikely that the women in these studies were aware of any potential link between dietary GI or GL and cancer at the time of participation, which ranged between the 1980s and 1990s.

Our meta-analysis of GI and GL intake and endometrial cancer risk does have limitations. The overall results are based on only five studies, and only four studies are included in the analysis stratified by BMI. However, all studies incorporated a large number of cases, particularly the EPIC study, which is a multicentre investigation of the association between diet and cancer risk in 10 European countries. Potential confounders such as parity, hormonal use, age at menarche and age at menopause were not universally adjusted for, which is not ideal when combining results; however, age, energy intake and BMI were adjusted for in all studies. We only compared low GI (GL) intake with high GI (GL) intake in reported categorisations, which differed from study to study. It is also difficult to determine with certainty any effect of publication bias or heterogeneity in such a small sample of studies, as indicated by the wide confidence intervals shown in the *I*^2^ test for heterogeneity. In addition, the possibility of residual confounding cannot be ruled out.

Future well-designed studies or consortium-based analyses with a large number of cases, and consequently, more power should be conducted, particularly for the examination of interactions in high-risk insulin-resistant population groups, that is overweight, obese and sedentary women, to confirm our results. Further research is required on the effects of a high GI or GL diet in ovarian cancer, as our systematic review included only two studies, both of which showed results similar to those seen for endometrial cancer risk.

In conclusion, consuming a high GL diet is associated with an increased risk of endometrial cancer and risk is further increased in obese women. Dietary GI does not appear to be related to endometrial cancer risk. Further research is required on GI, GL and ovarian cancer risk.

## Figures and Tables

**Table 1:** Characteristics of studies included in systematic review of dietary glycaemic index, glycaemic load and risk of endometrial and ovarian cancer

								**Adjusted confounders**												
**Study–year–location**	**Study design (mean follow-up)**	**Cases**	**Controls/cohort size**	**Diet assessment**	**Quality scale score**	**Median GI (IQ range)**	**Median GL (IQ range)**	**Age**	**BMI**	**Energy**	**Hormon.**	**Reprod.**	**Menstr.**	**Smoking**	**PA**	**Education**	**Alcohol**	**Fibre**	**Diabetes**	**Height**
*Endometrial*																				
Cust *et al* (2007a) Europe	Prospective cohort (6.4 years)	710	288 428	Self-reported/interviewed FFQ and 24-h recall	9/9	56 (53–58)	117 (94–144)	✓	✓	✓	#	#	#	✓	✓	#			#	✓
Larsson *et al* (2007) Sweden	Prospective cohort (15.6 years)	608	66 651	Self-reported 67-item FFQ, 96-item FFQ	9/9	80 (74–86)	181 (155–210)	✓	✓	✓	✓	✓	✓	#	#	✓			#	
Silvera *et al* (2005) Canada	Prospective cohort (16.4 years)	426	34 391	Self-reported 86-item FFQ	9/9	73 (67–77)	148 (125–169)	✓	✓	✓	✓	✓	✓	✓	✓		✓			
Folsom *et al* (2003) United States	Prospective cohort (−)^a^	415	23 335	Self-reported 126-item FFQ	9/9	85 (81–89)	170 (147–193)	✓	✓	✓	✓		✓	✓			✓		✓	
Augustin *et al* (2003a) Switzerland/Italy	Hospital-based case–control	410	753	Interviewed 37-item FFQ	6/9	82^1^ 74^2^ (70–88)	143^1^ 112^2^ (108–214)	✓	✓	✓	✓					✓			✓	
																				
*Ovarian*																				
Silvera *et al* (2007) Canada	Prospective cohort (16.4 years)	264	48 776	Self-reported 86-item FFQ	9/9	77 (63–92)	148 (125–169)	✓	✓	✓	✓	✓	✓		✓		✓	✓		
Augustin *et al* (2003b) Italy	Hospital-based case–control	1031	2411	Interviewed 78-item FFQ	6/9	74 (70–78)	185 (147–234)	✓		✓	✓	✓	✓		✓	✓	✓	✓	✓	

FFQ=Food Frequency Questionnaire; GI=glycaemic index; GL=glycaemic load; IQ=Inter-quartile.

Adjusted confounders: age; BMI=body mass index; Energy=energy intake; Hormon.=hormone replacement therapy/oral contraceptive use; Reprod.=reproductive factors, e.g., parity, age at first birth; Menstr.=menstrual history, e.g., age at menarche or menopause, menopausal status; Smoking; PA=physical activity; Education; Alcohol intake; Fibre=Fibre intake; Diabetes=History of diabetes.^#^Indicates potential confounders that were tested but not included in the final model.

a Total follow-up length 304 558 women-years. ^1^Values from Swiss centre, ^2^Values from Italian centre.
